# Accumulation Pattern of Amygdalin and Prunasin and Its Correlation with Fruit and Kernel Agronomic Characteristics during Apricot (*Prunus armeniaca* L.) Kernel Development

**DOI:** 10.3390/foods10020397

**Published:** 2021-02-11

**Authors:** Ping Deng, Bei Cui, Hailan Zhu, Buangurn Phommakoun, Dan Zhang, Yiming Li, Fei Zhao, Zhong Zhao

**Affiliations:** 1 Key Comprehensive Laboratory of Forestry, College of Forestry, Northwest A&F University, Shaanxi Province, Yangling 712100, China; d_p@nwsuaf.edu.cn (P.D.); cuibei@nwsuaf.edu.cn (B.C.); zhuhailan@nwsuaf.edu.cn (H.Z.); Buangurn@nwsuaf.edu.cn (B.P.); zhangdan0912@nwafu.edu.cn (D.Z.); lyming@nwsuaf.edu.cn (Y.L.); 2 College of Biology and Pharmacy, Yulin Normal University, Yulin 537000, China; 3 Beijing Agricultural Technology Extension Station, Beijing 100029, China; zhaofeifox@gmail.com

**Keywords:** apricot (*Prunus armeniaca* L.) kernel, amygdalin, cyanogenic glycoside, accumulation pattern, fruit quality

## Abstract

To reveal the accumulation pattern of cyanogenic glycosides (amygdalin and prunasin) in bitter apricot kernels to further understand the metabolic mechanisms underlying differential accumulation during kernel development and ripening and explore the association between cyanogenic glycoside accumulation and the physical, chemical and biochemical indexes of fruits and kernels during fruit and kernel development, dynamic changes in physical characteristics (weight, moisture content, linear dimensions, derived parameters) and chemical and biochemical parameters (oil, amygdalin and prunasin contents, β-glucosidase activity) of fruits and kernels from ten apricot (*Prunus armeniaca* L.) cultivars were systematically studied at 10 day intervals, from 20 days after flowering (DAF) until maturity. High variability in most of physical, chemical and biochemical parameters was found among the evaluated apricot cultivars and at different ripening stages. Kernel oil accumulation showed similar sigmoid patterns. Amygdalin and prunasin levels were undetectable in the sweet kernel cultivars throughout kernel development. During the early stages of apricot fruit development (before 50 DAF), the prunasin level in bitter kernels first increased, then decreased markedly; while the amygdalin level was present in quite small amounts and significantly lower than the prunasin level. From 50 to 70 DAF, prunasin further declined to zero; while amygdalin increased linearly and was significantly higher than the prunasin level, then decreased or increased slowly until full maturity. The cyanogenic glycoside accumulation pattern indicated a shift from a prunasin-dominated to an amygdalin-dominated state during bitter apricot kernel development and ripening. β-glucosidase catabolic enzyme activity was high during kernel development and ripening in all tested apricot cultivars, indicating that β-glucosidase was not important for amygdalin accumulation. Correlation analysis showed a positive correlation of kernel amygdalin content with fruit dimension parameters, kernel oil content and β-glucosidase activity, but no or a weak positive correlation with kernel dimension parameters. Principal component analysis (PCA) showed that the variance accumulation contribution rate of the first three principal components totaled 84.56%, and not only revealed differences in amygdalin and prunasin contents and β-glucosidase activity among cultivars, but also distinguished different developmental stages. The results can help us understand the metabolic mechanisms underlying differential cyanogenic glycoside accumulation in apricot kernels and provide a useful reference for breeding high- or low-amygdalin-content apricot cultivars and the agronomic management, intensive processing and exploitation of bitter apricot kernels.

## 1. Introduction

Apricot (*Prunus armeniaca* L.), which is an important Rosaceae family fruit crop, is a multipurpose tree species with ecological and economic value. It shows strong adaptation to stress and can be cultivated on marginal land. The kernels of apricot contain substantial amounts of oil [1], proteins, fiber, phenolics, minerals and bioactive compounds [2,3,4,5,6,7,8,9], and play an important role not only in human nutrition and health [10], but also in the medicine, food and cosmetic industries [11,12] and biodiesel [13,14,15]. Apricot kernels contain the toxic phenylalanine-derived cyanogenic glycoside amygdalin, accompanied by minor amounts of prunasin, which is a precursor of the diglucoside amygdalin, a β-D-monoglucoside of R-mandelonitrile. Upon tissue disruption, amygdalin and its precursor prunasin are degraded by specific β-glucosidases, resulting in the release of toxic hydrogen cyanide, which serves as a defense mechanism against generalist herbivores [16] and imposes a large constraint on the use of apricot kernels to provide human or animal nutrition because of their bitter taste and toxicity. The toxicity of cyanide is largely attributed to the cessation of aerobic cell metabolism by reversibly binding to the mitochondrial cytochrome oxidase a3 [17,18,19]. Clinical symptoms of acute cyanide poisoning, which usually occur less than 1 min after inhalation and within a few minutes after ingestion, include rapid respiration, a drop in blood pressure, rapid pulse, headache, dizziness, vomiting, abdominal pain, diarrhea, mental confusion, stupor, blue discoloration of the skin due to lack of oxygen (cyanosis), twitching and convulsions to coma and death [19,20,21]. A series of poisoning cases (adults, children, livestock) has been reported from the ingestion of bitter apricot kernels [21,22,23,24,25,26,27,28]. Long-term exposure to sublethal concentrations of cyanogenic glycosides can present ongoing health issues, such as Konzo, an irreversible motor neuron disease with clinical signs including the inability to walk, limited arm movement, and speech difficulties [29,30]. This poses an ongoing risk that needs to be managed to avoid future poisoning incidents. The bitter taste of apricot kernels is one of the main quality attributes of apricot cultivars, and has long been attributed to high accumulated levels of cyanogenic glycosides. Therefore, the use of apricot kernels for the development of drugs, food supplements, and functional foods may be of greater interest in markets where there are high-glycosides or non- or weakly cyanogenic varieties.

A large number of studies have been carried out to characterize the quality, composition and biochemistry of apricots [31,32,33,34,35,36,37]. However, all of these studies were focused on mature kernels in the harvesting stage. In addition, different hypotheses regarding the inheritance of the bitter/sweet trait of apricot kernels have been proposed [38,39], but no definitive model has been demonstrated. In particular, the processes involved in the metabolism and accumulation of cyanogenic glycosides in apricot kernels have not been well defined [40,41,42,43]. Furthermore, no global assessment of fruit quality attributes and cyanogenic glycoside accumulation patterns in apricot kernels, or their relationships with each other in different apricot cultivars and stages of fruit development, has been systematically reported. The elucidation of cyanogenic glycoside accumulation patterns in different apricot cultivars at different developmental stages will greatly enhance the understanding of the genetic differences between sweet and bitter apricot cultivars, and such knowledge is an essential prerequisite for further research leading to the modification of amygdalin contents in apricot kernels. On the side, understanding the enzymes and mechanisms involved in the accumulation of cyanogenic glycosides and the importance of this pathway in physiological processes and plasticity will provide a rationale for regulating the contents of cyanogenic glycosides in apricot kernels. Amygdalin and prunasin are hydrolyzed by specific enzymes called β-glucosidases. For the reasons mentioned above, in this study, we monitored physical parameters (weight, moisture content, linear dimensions and derived relative parameters) and chemical parameters (oil content, amygdalin and prunasin and catabolic enzyme β-glucosidase activity) in the fruits and kernels of ten apricot cultivars at intervals of 10 days, from 20 days after 50% of flowering on the tree (DAF) until maturity. The aims were to analyze the accumulation pattern of cyanogenic glycosides and to preliminarily explore the associations between cyanogenic glycoside accumulation and agronomic characteristics linked to the fruit and kernel quality during fruit and kernel development. Based on the accumulation pattern of cyanogenic glycosides and these associations, we could further understand the metabolic mechanisms underlying the differential accumulation of cyanogentic glycosides and conduct further research on their regulatory mechanism in bitter apricot kernels; this could provide a useful reference for further developing more effective methodologies for breeding high- or low-amygdalin-content apricot cultivars, and help orchardists optimize the management of the nutritional potential of apricot fruit and harvest time.

## 2. Materials and Methods

### 2.1. Plant Materials and Sample Collection

Ten apricot cultivars were used as the test material in this study, including seven bitter-kernelled cultivars (Shanku-1 (SK-1), Cuanzhihong (CZH), Jiguang (JG), Jidan (JD), Yangshao (YS), Qiuhong (QH), Daguo (DG)) and three sweet-kernelled cultivars (Shantian-1 (ST-1), Weixuan-1 (WX-1), Zhengkui (ZK)). All assayed apricot cultivars were grown in an experimental apricot orchard at Weihe Experiment Station located in Zhouzhi County, Shannxi Province, China (N 34°16′27.08″, E 108°5′2.98″). All experimental trees (diameter at breast height (16–18 cm), tree height (4.5–7 m), crown width (3.5–4.5 m)) grown under the same standard agronomic management regime were planted with 3 × 4 m spacing in 1998 in rows in a north-south orientation. Five trees from each cultivar that showed similar growth and no plant pests or diseases were selected. The main phenological stages were noted weekly until flowering. A total of 60 healthy developing fruits from each tree of each apricot cultivar were manually picked from four different aspects (15 fruits in each aspect (east, south, west and north)) in the external canopy from positions without shading by leaves to ensure adequate light acquisition (a total of 300 fruits per cultivar per harvest period); collection began at 20 DAF and continued at 10-day intervals until full maturity (10 April to 22 June 2019, about 90 days). Flowering time was defined as the date when 50% of flowers on the tree were open. Fruit maturity was determined according to appearance, fruit peel color, flesh firmness, and taste.

The fruits of each cultivar were collected and pooled in polythene bags, placed in an ice box, and then transferred to the laboratory for phenotypic analysis at each harvest date. Extreme caution was taken to avoid collecting fruits with signals of fungal or bacterial infestation. Since the endocarp of the fruit began to lignify at approximately 30 DAF, after which seed growth increased, all parameters before 30 DAF were derived from the whole fruits, whereas after 30 DAF, the fruits were dissected into two portions: the fleshy mesocarp (including ectocarp) and kernels (Figure S1), which were processed separately. The fruits were cracked and shelled manually, and the kernels were separated from the shell (endocarp), taking care to not damage the kernels. Only apricots with normal kernels were used, and shriveled kernels were discarded.

### 2.2. Determination of Pomological Characteristics of Apricot Fruit and Kernels

Twenty fresh fruits and fresh kernels of each cultivar were randomly sampled from 300 fruits at each harvest period in triplicate and measured separately to determine fresh fruit weight (FWt) and kernel weight (KWt) using an electronic analytical digital scale balance (GT 480, Ohaus, Korea) with 0.001 g sensitivity. Three linear dimensions, as length, width and thickness (Figure S2), were measured with a digital Vernier caliper (SF2000, Guilin Guanglu Measuring Instrument Co. Ltd., Guilin, China) with an accuracy of 0.01 mm. On the basis of the three linear dimensions, other parameters, including shape index (SI), geometric mean diameter (GMD), sphericity (Sph) and surface area (SA) [44,45,46], were transformed and calculated with the following equations: SI = (W + T)/2L, GMD (mm) = (L × W × T)^1/3^, Sph = GMD/L, SA (cm^2^) = π × GMD^2^ (where L is length, W is width, and T is thickness). The water content of the kernels (%) was determined as a percentage of fresh kernel weight by weighing the kernels before and after drying to constant weight in a cryogenic vacuum freeze-drying apparatus (133 × 10^−3^ mBar, −60 °C, 48 h) as follows: kernel water content (%) = (fresh weight (g) − dry weight (g)) × 100/fresh weight (g).

### 2.3. Extraction and Determination of Apricot Kernel Oil

Lyophilized apricot kernels from each cultivar and each sampling date were pulverized with a universal grinder (XM-2500, Xuman, Zhejiang, China) and screened with a 60-mesh sieve. Lipids were extracted from apricot kernel powder in triplicate with 1:20 (vol/vol) petroleum ether (b.p. 30–60 °C) as the solvent by using a Soxhlet apparatus for 8 h. The remaining solvent was removed by a rotary vacuum evaporator at 40 °C, and drying was conducted with a gentle stream of nitrogen to yield the apricot kernel oil. The yield of the oil was expressed as a percentage of dry weight as follows: oil content (%) = (initial weight of lyophilized kernel powder (g) − final weight of defatted powder (g)) × 100/initial weight of lyophilized kernel powder (g).

### 2.4. Identification and Quantification of Amygdalin and Prunasin in Apricot Kernels

For the extraction of amygdalin and prunasin, 10.0 g of apricot kernel powder was defatted with 200 mL of petroleum ether for 8 h using a Soxhlet apparatus; then 0.15 g of defatted powder (if the kernel was bitter) and 0.3 g (sweet kernel) were homogenized in 50 mL of methanol. The suspension was placed in an ultrasonic bath at 30 °C for 30 min, and then centrifuged at 10,000× *g* for 15 min. The supernatant was immediately filtered through a 0.22 μm organic membrane filter with a syringe for high-performance liquid chromatography (HPLC) analysis. HPLC analysis was performed using an Agilent 1260 system (1260 INFINITY II, Agilent, Santa Clara, CA, USA), and chromatographic separation was carried out using an RP18 stainless-steel column 250 × 4.6 mm i.d. 5 μm XBridge^®^ C18 column (Waters, Milford, MA, USA) at a UV wavelength of 214 nm with 20% methanol as the mobile phase. The flow rate was isocratically controlled at 0.6 mL/min, and the column temperature was maintained at 30 °C. Peak identification was performed through comparison with the retention times of standard solutions. The average retention times for amygdalin and prunasin were 23.22 and 31.96 min, respectively. Cyanogenic glycosides were quantified via linear regression methods using an amygdalin (CAS:29883-15-6; 99%; Sigma-Aldrich, Shanghai, China) and prunasin (CAS:99-18-3; ≥96%; YuanYe Biotechnology, Shanghai, China) standard curve, and the results were expressed as milligrams of per gram dry apricot kernel weight. All samples were prepared in triplicate.

### 2.5. Evaluation of β-Glucosidase Activity in Apricot Kernels

The β-glucosidase activity was measured using specific enzyme activity assay kits (Suzhou Com in Biotechnology Co., Ltd., Suzhou, China). Fresh mesocarps and kernels were sliced into approximately 0.2–0.5 cm-wide segments, immediately frozen and pulverized in liquid N_2_. The enzyme solution was extracted from ground mesocarp and kernel samples. One hundred milligrams of powder (fresh weight (FW)) was homogenized in 1 mL extraction solution (50 mM phosphate buffer, pH 7.0) in an ice bath, and the mixture was then centrifuged at 15,000× *g* for 10 min at 4 °C. The supernatant was placed on ice, then we carried out the enzyme-catalyzed reaction according to the kit protocol. Finally, assays of enzyme activity were executed using Microplate reader in the 96-well microplate at room temperature (25 °C) at 405 nm. In all cases, one unit of β-glucosidase activity was defined as the production of 1 nmol *p*-nitrophenol per minute per g of tissue. Three replicates were performed for each measurement, and the results were expressed as specific activity (nmol/min/g FW).

### 2.6. Statistical Analysis

Data are reported as the means ± standard error of triplicate measurements. Significant differences among the samples were calculated using one-way ANOVA followed by the least significant difference method (LSD) at the 5% level with the SPSS (IBM SPSS Statistics 23, IBM SPSS Inc., Chicago, IL, USA) statistical software package. A correlation graph was generated via the Spearman method to resolve the correlation between the quality traits analyzed. Principal component analysis (PCA) was performed to evaluate the relationships among variables and any possible cultivar groupings, trends, or outliers using Origin statistical software.

## 3. Results

### 3.1. Changes in the Agronomic Characteristics of Apricot Fruit and Kernels at Different Developmental Stages

**Figure 1:**
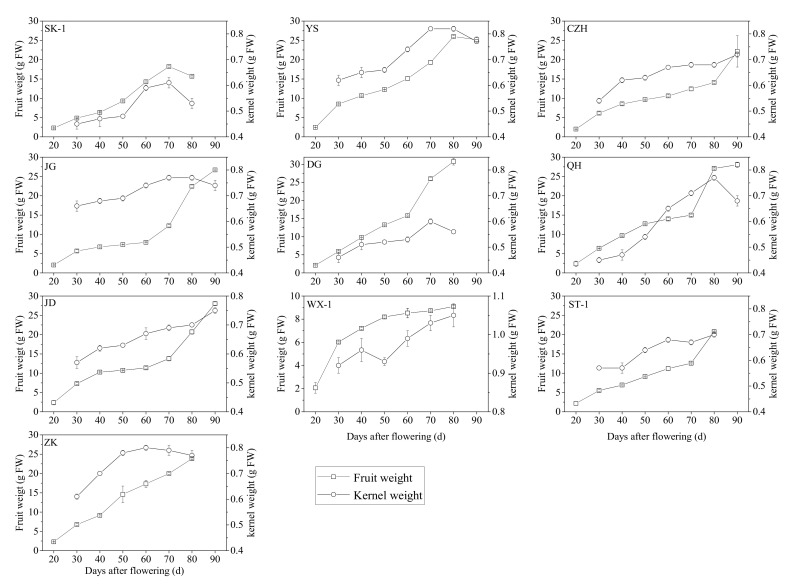
Changes in fresh weight during the development of fruits and kernels in ten apricot cultivars. “Days after flowering” means days after 50% of flowers on the tree were open. Values are means ± standard error (*n* = 3). FW, fresh weight; cultivars: CZH, “Chuanzhihong”; DG, “Daguo”; JD, “Jidan”; JG, “Jiguang”; QH, “Qiuhong”; SK−1, “Shankuyihao”; ST−1, “Shantianyihao”; WX−1, “Weixuanyihao”; YS, “Yangshao”; ZK, “Zhengkui”.

The results related to the linear dimensions and other agronomic characteristic parameters of the tested apricot fruits and kernels are presented in Table S1. High variability in all studied quality attributes was found among the set of evaluated apricot cultivars and at different ripening stages. The single-fruit weight (FWt) ranged from 2.02 ± 0.03 g to 30.78 ± 0.96 g in all tested varieties throughout the fruiting stage, and the differences in single-fruit weight among genotypes were highly significant (Table S1). The fresh fruit weight rapidly and linearly increased with fruit growth and maturity to reach the highest values in “DG” (30.78 g), “YS” (25.99 g), “ZK” (23.83 g), “ST-1” (20.79 g) and “WX-1” (9.09 g) at 80 DAF, and “QH” (28.03 g), “JD” (28.03 g), “JG” (26.68 g) and “CZH” (22.14 g) at 90 DAF, after which it decreased slightly to 25.16 g in “YS” and 15.17 g in “SK-1” at complete maturity (Table S1, Figure 1). Kernel weight accumulation followed a similar pattern in the ten varieties during kernel development, ranging from 0.45 ± 0.01 g to 1.05 ± 0.03 g (Table S1). Kernel weight (KWt) differed significantly among the different apricot genotypes and different ripening stages. The highest kernel weight was found in the “WX-1” genotype (1.05 g) in the complete maturity stage (Figure 1).

As shown in Table S1 and Figures S3–S6, the linear dimensions (W, L, T), GMD, SA, Sph, and SI of the fruits and kernels varied significantly among the tested apricot varieties and ripening stages. The quality parameters of each cultivar increased gradually with the development of fruits and kernels and reached their highest values at full ripening (80 or 90 DAF). The maximum values in fruit were found in “JD” for L (39.68 mm), “YS” for W (40.31 mm), “DG” for T (40.69 mm), “QH” for GMD (39.15 mm) and SA (4819.03 mm^2^), “DG” for Sph (1.07) and SI (1.11), and those in kernels were found in “WX-1” for L (20.52 mm), W (14.38 mm), GMD (13.72 mm) and SA (591.78 mm^2^), and “YS” for T (8.95 mm), Sph (0.78) and SI (0.70), respectively. “WX-1” was the cultivar showing the lowest values of these quality parameters for fruit at full ripening (80 DAF). For kernels at full maturity, “SK-1” was the cultivar with the lowest T, GMD and SA values. “ST-1” exhibited the lowest W, Sph and SI values, and the smallest L value was found in “DG”.

### 3.2. Changes in the Total Lipid Content and Moisture Content during Apricot Kernel Maturation

**Figure 2:**
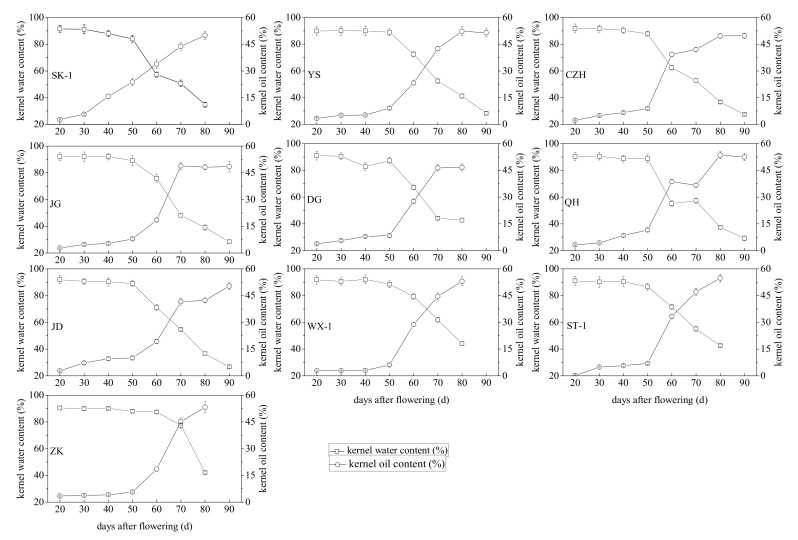
Changes in water and oil contents (%) during kernel development in ten apricot cultivars. Kernel water content of the kernels (%) was determined as a percentage of fresh kernel weight by weighing the kernels before and after drying to constant weight. Oil content was expressed as a percentage of dry kernel weight. Values are means ± standard error (*n* = 3). cultivars: CZH, “Chuanzhihong”; DG, “Daguo”; JD, “Jidan”; JG, “Jiguang”; QH, “Qiuhong”; SK−1, “Shankuyihao”; ST−1, “Shantianyihao”; WX−1, “Weixuanyihao”; YS, “Yangshao”; ZK, “Zhengkui”.

During apricot kernel development, kernel oil accumulation followed a similar sigmoid pattern in most of the tested apricot varieties, although there was some variability in the total lipid content among the ten tested apricot cultivars at different ripening stages (Figure 2). The oil yields from these kernels varied from 0.1 to 54.81% at all stages of development, and the “ST-1” cultivar showed a higher rate of lipid accumulation (54.81%). The accumulation of oil in the developing apricot kernel started at the beginning of seed development and was very slow from 20 to 50 DAF; then, it showed a significant linear increase with kernel development in most of the tested apricot varieties and reached a maximum level at 70 DAF in “JG” and “DG” and 80 DAF in “YS”, “CZH”, “WX-1”, “ST-1” and “ZK”; thereafter, it remained stable or decreased slightly in “YS”, “CZH”, “JG” and “DG” until full maturity. In the “YS”, “CZH”, “WX-1”, “ST-1” and “ZK” cultivars, the amount of accumulated oil varied from 9.11 to 52.29%, 8.92 to 49.66%, 6.06 to 53.07%, 6.98 to 54.81%, and 5.71 to 53.31% from 50 to 80 DAF, respectively, while in the “JG”, “DG” and “JD” cultivars, it changed from 7.87 to 48.12%, 8.40 to 46.40% and 10.04 to 41.59% from 50 to 70 DAF, respectively. The maximum oil content in all tested cultivars was observed in “ST-1” (54.81%) at full ripening (80 DAF). Among the tested apricot varieties, oil accumulation in the “SK-1” variety showed a linear increase from the beginning of kernel development to full maturity (2.85 to 49.97%), while in varieties “WX-1”, “ST-1” and “ZK”, oil accumulation increased rapidly from 50 DAF to full maturity (80 DAF). In contrast, oil accumulation in the “QH” variety displayed a double sigmoid pattern, with marked increases from 50 to 60 DAF and 70 to 80 DAF during kernel development. The moisture content of the kernels showed an opposite pattern to kernel oil accumulation throughout the development process. The pattern of kernel moisture content presented a slight reduction from 20 to 50 DAF, then a rapid decrease from 50 DAF to full maturity for all tested apricot cultivars and reached the lowest levels of 34.72% in “SK-1”, 28.32% in “YS”, 27.42% in “CZH”, 28.34% in “JG”, 42.76% in “DG”, 29.22% in “QH”, 26.68% in “JD”, 44.18% in “WX-1”, 42.60% in “ST-1” and 42.13% in “ZK” at the end of the sampling (80 or 90 DAF) (Figure 2).

### 3.3. Accumulation of Cyanogenic Glycosides during Apricot Kernel Development

**Figure 3:**
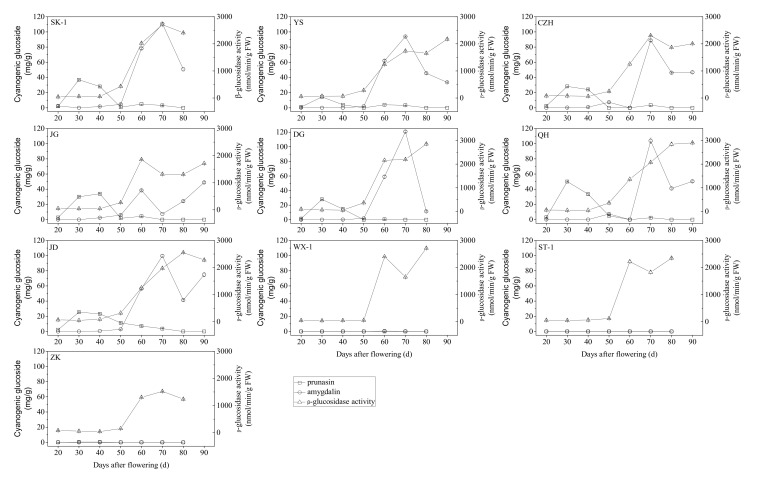
Changes in cyanogenic glycosides (mg/g DW) and β-glucosidase activity (nmol/min/g FW) during kernel development in ten apricot cultivars. Values are means ± standard error (*n* = 3). FW, fresh weight; DW, dry weight; cultivars: CZH, “Chuanzhihong”; DG, “Daguo”; JD, “Jidan”; JG, “Jiguang”; QH, “Qiuhong”; SK−1, “Shankuyihao”; ST−1, “Shantianyihao”; WX−1, “Weixuanyihao”; YS, “Yangshao”; ZK, “Zhengkui”.

The dynamic changes in cyanogenic glycoside concentrations were determined by HPLC among the ten tested apricot cultivars at different developmental stages. The results showed that the levels of prunasin and amygdalin among different bitter kernel cultivars followed markedly different time-courses during fruit maturation (Figure 3). The quantification of amygdalin and prunasin contents in kernels showed that very low or undetectable contents were maintained in the sweet kernel cultivars (“WX-1”, “ST-1”, “ZK”) throughout the development of the kernel. Prunasin accumulation began before 20 DAF in all tested bitter-kernelled genotypes, after which the prunasin content significantly increased and reached the highest levels in “QH” (50.10 mg/g dry weight (DW)), “SK-1” (36.91 mg/g DW), “JG” (34.10 mg/g DW), “CZH” (28.15 mg/g DW), “DG” (27.93 mg/g DW), “JD” (25.47 mg/g DW), and “YS” (14.36 mg/g DW) at 30 DAF; thereafter, the prunasin content decreased markedly from 30 to 50 DAF and further declined to close to zero or was undetectable throughout the remainder of the developmental phases. However, the amygdalin accumulation pattern in kernels was different from that of prunasin, both within and among all the studied bitter kernel cultivars and different developmental stages, since during the early stages of apricot fruit development (before 50 DAF), amygdalin was present in bitter kernels in quite small amounts or was undetectable (ranging from 0 to 7.38 mg/g DW), and its content was significantly lower than the content of prunasin. Subsequently, amygdalin levels increased significantly after 50 or 60 DAF and was highest at 70 DAF (128 mg/g DW in “DG”, 110.84 mg/g DW in “SK-1”, 103.85 mg/g DW in “QH”, 99.62 mg/g DW in “JD”, 93.87 mg/g DW in “YS”, and 88.76 mg/g DW in “CZH”), except in the “JG” cultivar, which showed the first peak level at 60 DAF (38.53 mg/g DW). Thereafter, amygdalin content decreased dramatically in “SK-1” (50.71 mg/g DW), “DG” (11.43 mg/g DW), “YS” (33.49 mg/g DW) and “CZH” (46.15 mg/g DW) from 70 DAF to full maturity (80 DAF or 90 DAF), while the amygdalin contents of “QH” and “JD” decreased to 41.01 mg/g DW and 41.37 mg/g DW, respectively, from 70 to 80 DAF, and then increased slightly until full maturity (90 DAF). It is worth mentioning that the accumulation pattern of amygdalin in variety “JG” was different from that in the other varieties, as “JG” showed the first peak (38.53 mg/g DW) at 60 DAF, followed by a decrease to 7.44 mg/g DW at 70 DAF, and an increase to the maximum level (48.95 mg/g DW) at full maturity (90 DAF).

### 3.4. β-Glucosidase Activity during Apricot Kernel Development

β-glucosidase activity in the kernels of all tested apricot cultivars was analyzed with enzyme activity assay kits throughout the development process. High variability in β-glucosidase activity was found among the ten tested apricot cultivars at different ripening stages (Figure 3). During the early stages of apricot kernel development (20 to 40 DAF), β-glucosidase activity in the kernels of all tested apricot cultivars remained quite low, ranging from 36.68 to 101.38 nmol/min/g FW, which was followed by a trend of a slow increase in “SK-1”, “YS”, “CZH”, “JG”, “DG”, “QH” and “JD” from 40 to 50 DAF, while “WX-1”, “ST-1” and “ZK” maintained relatively low levels. Subsequently, β-glucosidase activity increased significantly, beginning at 50 DAF, and reached its highest levels in “JG” (1855.17 nmol/min/g FW), “WX-1” (2398.92 nmol/min/g FW) and “ST-1” (2218.06 nmol/min/g FW) at 60 DAF, in “SK-1” (2708.24 nmol/min/g FW), “CZH” (2305.48 nmol/min/g FW), “ZK” (1523.22 nmol/min/g FW) and “YS” (1739.82 nmol/min/g FW) at 70 DAF, and in “DG” (nmol/min/g FW), “QH” (2837.74 nmol/min/g FW) and “JD” (2550.32 nmol/min/g FW) at 80 DAF. Thereafter, β-glucosidase activity decreased in cultivars “SK-1”, “JD” and “ZK” and increased in cultivars “DG” and “QH” until full maturity, while it first decreased in cultivars “WX-1”and “ST-1” until 70 DAF and cultivars “YS”, “CZH” and “JG” until 80 DAF, and then increased as the fruit gradually matured.

### 3.5. Correlation Analysis

**Figure 4:**
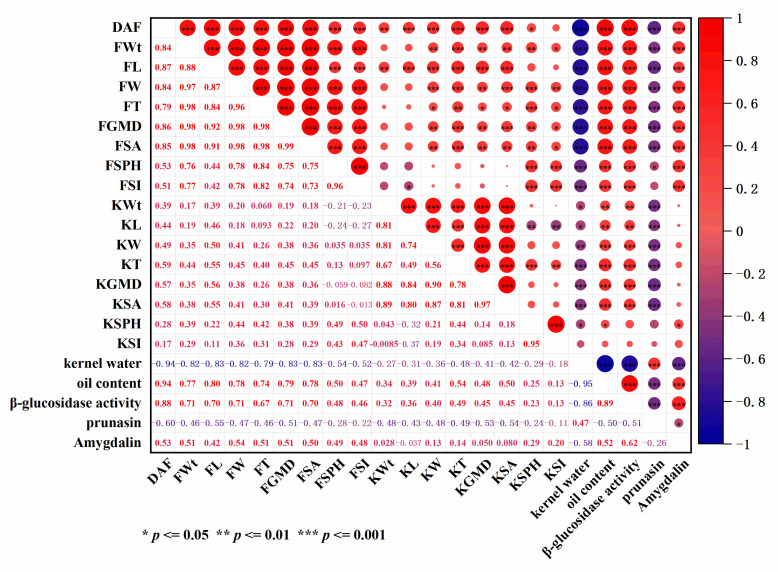
Correlation among physical, chemical and biochemical indexes and cyanogenic glycoside accumulation in ten apricot cultivars during fruit and kernel development. Days after flowering, DAF; fresh fruit weight, FWt; fruit length, FL; fruit width, FW; fruit thickness, FT; fruit geometric mean diameter, FGMD; fruit surface area, FSA; fruit sphericity, FSPH; fruit shape index, FSI; fresh kernel weight, KWt; kernel length, KL; kernel width, KW; kernel thickness, KT; kernel geometric mean diameter, KGMD; kernel surface area, KSA; kernel sphericity, KSPH; kernel shape index, KSI.

To search for possible links between the examined physical, chemical and biochemical indexes and cyanogenic glycoside compound accumulation in the fruits and kernels of 10 apricot cultivars during different developmental stages, Spearman’s correlation analysis was performed. As seen in Figure 4, significant correlations were observed for some apricot fruit and kernel physical, chemical and biochemical attributes among different cultivars and ripening stages. FWt showed significantly positive correlations with linear dimensions (L, W, T) and their derived parameters (GMD, SA, SPH) in fruits and kernels, except for KWt, kernel length (KL) and kernel shape index (KSI), which exhibited weak relationships, while the relationships of the contents of kernel water and prunasin with most of the other remaining measured parameters were significantly negative. There was a positive correlation between kernel oil content and the physical, physiological and biochemical parameters of fruits and kernels, especially the pomological parameters of the fruit (*r* = 0.74 to 0.80), and a significantly negative correlation between kernel oil content and kernel moisture content (*r* = −0.95). Similar correlations were observed for β-glucosidase activity, which was highly negatively correlated with kernel moisture content (*r* = −0.85) and significantly positively correlated with kernel oil content (*r* = 0.89). However, the correlations between the content of amygdalin in kernels and most of the tested indexes showed an opposite accumulation pattern to the content of prunasin during maturity. The amygdalin contents of kernels presented a positive correlation with kernel oil content (*r* = 0.52), β-glucosidase activity (*r* = 0.62), and fruit dimension parameters, but exhibited no or weak positive correlation with those of kernels.

### 3.6. Principal Component Analysis (PCA)

**Figure 5:**
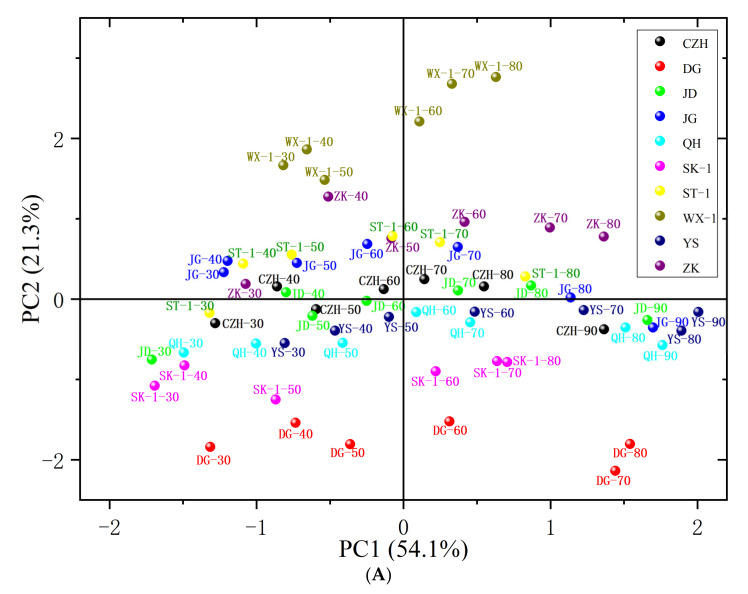

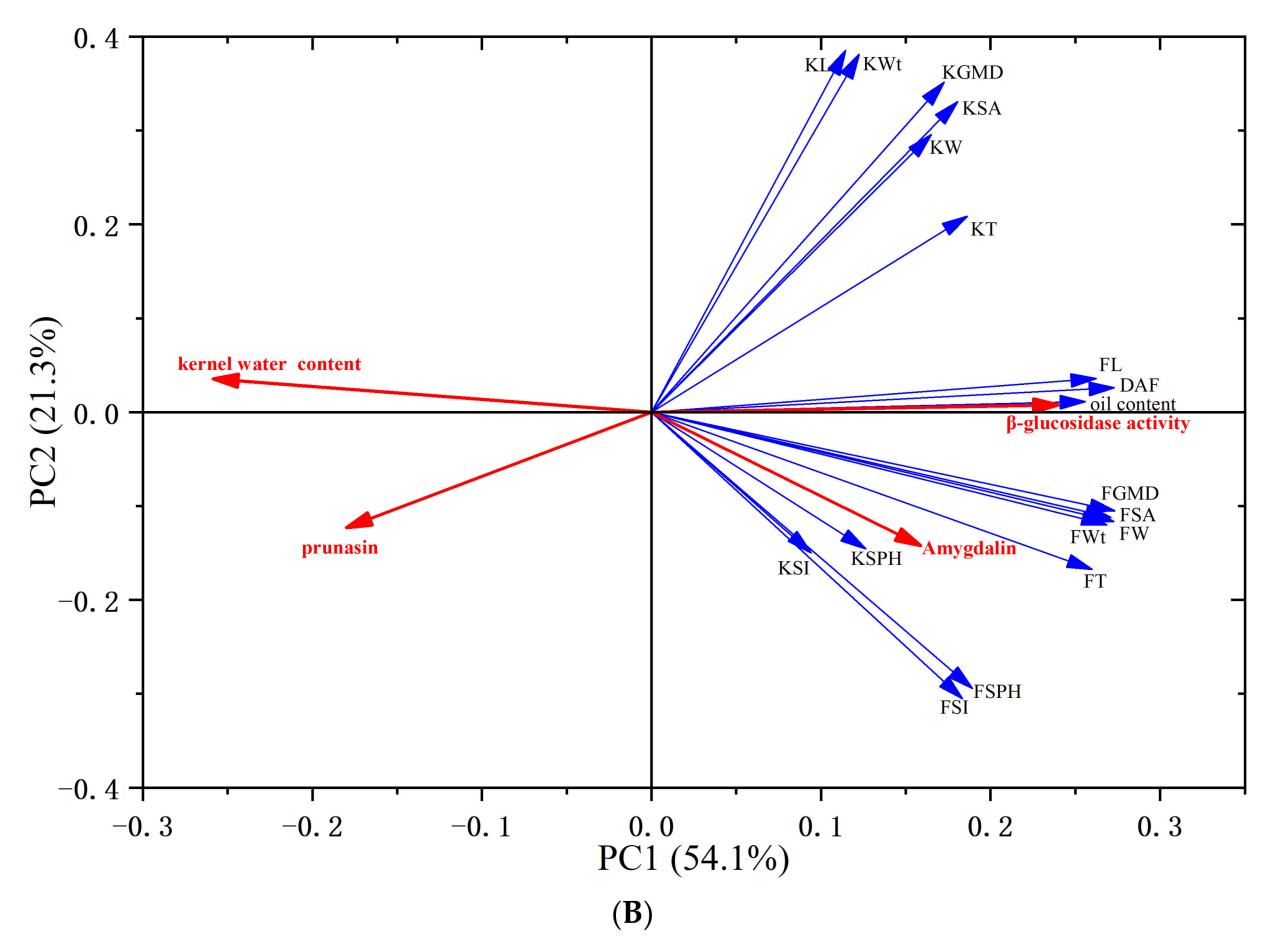
Principal component analysis (PCA) score plot (**A**) and loading plot (**B**) of ten apricot cultivars at different developmental stages. Numbers (30~90) after the cultivars in score plot (**A**) represent the days after flowering (DAF). Different color solid spheres in score plot (**A**) represent different cultivars: CZH, “Chuanzhihong”; DG, “Daguo”; JD, “Jidan”; JG, “Jiguang”; QH, “Qiuhong”; SK-1, “Shankuyihao”; ST-1, “Shantianyihao”; WX-1, “Weixuanyihao”; YS, “Yangshao”; ZK, “Zhengkui”. Blue and red arrows represent the tested physical, chemical and biochemical indexes: days after flowering, DAF; fresh fruit weight, FWt; fruit length, FL; fruit width, FW; fruit thickness, FT; fruit geometric mean diameter, FGMD; fruit surface area, FSA; fruit sphericity, FSPH; fruit shape index, FSI; fresh kernel weight (KWt); kernel length (KL); kernel width (KW); kernel thickness, KT; kernel geometric mean diameter, KGMD; kernel surface area, KSA; kernel sphericity, KSPH; kernel shape index, KSI.

To obtain an overview of the internal structure of the relationships among cyanogenic glycosides and the physical, physiological and biochemical indexes of fruits and kernels among different apricot cultivars and ripening stages, PCA (Table 1, Figure 5) was carried out in our work, which allows us to detect and interpret the sample patterns and their similarities and differences. Thus, the initial data matrix (containing all tested indexes of fruits and kernels among different apricot cultivars and ripening stages) is projected onto a smaller number of variables called principal components by PCA to convert into a score matrix (apricot cultivars and development stages) and loading matrix (all tested indexes). In this way, the quantification of the amount of useful information is gathered into a model that is easier to interpret than the original data set. In this study, it showed that more than eighty percent of the observed variability was explained by the first three principal components. The first, second, and third principal components (PCs) with eigenvalues >1 accounted for 54.07%, 21.32% and 9.17%, respectively, of the total variability. The cumulative contribution rate reached 84.56% (Table 1).

Table 2 shows the correlations between the original variables and the first three principal components. PC1 mainly represented not only fruit physical attributes (fruit geometric mean diameter (FGMD), DAF, fruit width (FW), fruit surface area (FSA), and FWt), but also the amygdalin and prunasin accumulation pattern; PC2 mainly explained KL, KWt, kernel geometric mean diameter (KGMD), kernel surface area (KSA) and kernel width (KW); PC3 mainly represented KSI, kernel sphericity (KSPH) and kernel thickness (KT). Most of the tested indexes, except for kernel water content and prunasin activity, were positively correlated with PC1, while most kernel physical attributes were positively correlated with PC2 (Table 2, Figure 5B).

Scores and loading plots of the first two PCs obtained from all tested indexes of fruits and kernels among different apricot cultivars and ripening stages are presented in Figure 5. According to the PCA score plot (Figure 5A), all tested samples were distributed in different quadrants for different apricot cultivars and different developmental stages of fruits and kernels, and especially “WX-1”, “DG”, “ZK”, and “SK-1” were seen well distinguished from other samples. The PCA loading plot (Figure 5B) shows the extent and nature of each tested index contribution to the principal components. The first PC not only revealed differences in amygdalin and prunasin contents and β-glucosidase activity among genotypes in different developmental stages, but also distinguished between fruit physical attributes. Negative values for PC1 indicated cultivars with higher kernel water and prunasin contents at the early stages of apricot kernel development. Cultivars such as “CZH”, “JG” and “JD” belonged to this group. The higher positive values for PC1 corresponded to cultivars with high β-glucosidase activity, oil contents and fruit physical attributes (FGMD, FSA, FWt) at ripening stages (“YS”, “JD”, “JG”), as shown in Figure 5A. Proceeding from positive to negative values of PC2, the amygdalin and prunasin contents of apricot kernels showed an overall increase; as a result, the sweet and bitter kernel cultivars were clearly separated. The group of varieties with the lowest negative PC2 values (such as “DG”, “SK-1” and “QH”) stands out, especially due to their high amygdalin contents and low values of kernel physical attributes (KWt, KGMD, KSA) at each developmental stage.

## 4. Discussion

Secondary metabolism is generally restricted to specific stages of organism development and occurs in specialized tissue. In most species, the association between the dependence at a particular developmental stage and ecophysiological requirements indicates that the biosynthesis and accumulation of secondary metabolites are coordinately regulated in time and space. Fruit quality was the conjunction of physical and chemical characteristics which give good appearance and acceptability to the consumable product. Numerous pomological traits influence the fruit quality, among them, the fruit size, shape and taste are fundamental quality attributes. The secondary metabolite biosynthesis and accumulation and fruit pomological traits are correlated to geographical environment, cultivar, developmental stages, soil, climate, cultivation system, agronomic management, harvest time, and level of maturity. In this study, sampling fruits and kernels of ten apricot cultivars grown in the same environment under the same cultivation conditions and agricultural management regime could avoid the complex effect of genetic background, highlight common trends and reveal possible differential accumulation mechanisms of cyanogenic glycosides in bitter apricot kernels. These results could provide important information for bitter taste regulation in apricot kernels and consumer-oriented breeding and contribute to establishing quality criteria for selecting apricot genotypes for amygdalin production.

Agronomic characteristics must be taken into account in the study of active components. In this study, apricot fruits and kernels displayed high variability, giving rise to great diversity in the linear dimensions, (W, L, T), GMD, SA, Sph and ripening date; most of these pomological characteristics are of interest for improving quality traits in apricot breeding programs. The accumulation of fresh fruit weight and kernel weight followed a similar pattern in ten tested varieties during development, which increased with development and declined slightly in some cultivars at the final maturity stage (Figure 1). During fruit and kernel development, apricot exhibits the continuous synthesis and accumulation of organic acids, carbohydrates, protein and lipids [47], leading to increases in fruit and kernel weights, which compensates for the moisture loss from the fruits and kernels. Previous studies also reported high variability among apricot cultivars regarding the above fruit characteristics [48,49,50,51,52,53,54]. Of course, there were some differences that may be the result of the different ecogeographical groups of the studied cultivars between our results and those of the above authors. In addition, the quality parameters may not be independent of each other, and relationships among them should be studied to improve decision making regarding production objectives related to fruit quality by using a limited number of independent parameters in breeding programs and orchard management. Multivariate analysis methods enable the investigation and interpretation of the relationships between varieties [32,55,56,57,58]. In this study, most of the examined apricot fruit and kernel physical attributes showed significant correlations with each other in different cultivars and ripening stages (Figure 4). The contents of prunasin and moisture in kernels were negatively correlated with those in other tested indexes, but the content of amygdalin in kernels showed an opposite pattern. In the PCA, several principal components were used to reveal the internal structural relationships between pomological traits and cyanogenic glycoside accumulation, as well as to further support the correlation analysis. According to our results, all tested samples could be distinguished according to the cultivar, development stages and cyanogenic glycoside content in the PCA score plot. The cumulative contribution rate of the first three principal components reached 84.56%. In the PCA loading plot (Figure 5), the developmental stages were clearly separated by PC1, which explained 54.07% of the variance. During the early stages of apricot kernel development (from 20 to 50 DAF), kernel moisture and prunasin contents were major contributors to PC1. Kernel physical attributes and cyanogenic glycoside contents at different developmental stages of kernels were clearly separated by PC2, which explained 21.3% of the variance. These results were in agreement with the correlation analysis among the tested variables.

Apricot kernels are a rich source of oil, with great nutritional and medicinal value due to the presence of different saturated and unsaturated fatty acids and phenolic compounds. The study of oil accumulation during kernel development was important for determining the optimum period in which apricot kernels accumulate the maximum amount of oil. In our study, kernel oil accumulation exhibited a sigmoidal pattern with kernel development, similar to the seeds and kernels of other higher plants. The accumulation of oil in apricot kernels was very slow at the beginning of kernel development (from 20 to 50 DAF), and the lipids synthesized by immature apricot kernels may be used to develop new kernel tissues [59]. The most active period of oil accumulation occurred at 50 to 70 or 80 DAF, indicating that the optimal harvest time for obtaining the maximum oil content was at 70 or 80 DAF for most tested apricot varieties. The dramatic increase in total oil content could be explained not only by the fact that the fruits and kernels were nearly completely formed in this period, which would favor the synthesis and accumulation of reserve lipids, but also by the loss of kernel moisture (Figure 1). The flattening of or reductions in oil accumulation observed during the last stage of maturity in “YS”, “CZH”, “JG” and “DG” could be caused by continued dry matter accumulation after oil accumulation ceased or slowed down. The values of these parameters obtained in this study were slightly lower than those reported previously [2,9,60,61,62], possibly due to differences in the cultivar, the environment or growing conditions. In addition, the apricot kernel oil content presented positive correlations with nearly all parameters of the fruits and kernels measured in this study, especially the pomological parameters of fruit (*r* = 0.74–0.80) and β-glucosidase activity (*r* = 0.89), but showed a significantly negative correlation with kernel moisture content (*r* = −0.95). There was little correlation between apricot kernel oil accumulation and amygdalin (*r* = 0.52). The temporal accumulation patterns of oil might be useful for evaluating apricot kernel quality, determining the optimal harvest period, and elucidating the molecular and metabolic mechanisms leading to increased oil biosynthesis and accumulation in apricot kernels, and may indicate that it might be possible to harvest some tested apricot varieties for oil before the current harvest time.

Bitterness in apricot is one of the most important traits studied by apricot growers and breeders, and is related to the content of amygdalin [42,63,64]. In the present study, two kinds of cyanogenic glycosides, amygdalin and prunasin, were confirmed in all bitter apricot kernels. The accumulated amygdalin and prunasin concentrations remained very low or were undetectable throughout the examined developmental stages in all non-bitter cultivars and were quite different among the bitter-kernelled apricot cultivars and at different developmental stages. Prunasin was mainly synthesized and accumulated during the early stages of apricot kernel development (20 to 50 DAF), after which its content declined to close to zero or was undetectable throughout the remainder of the developmental phases in all the studied bitter kernel cultivars. Although prunasin predominated in the early stages of apricot kernel development, the synthesis and accumulation of amygdalin during later phases, concomitant with a decline in prunasin levels, caused amygdalin to predominate at the maturity stage; thus, the accumulation pattern of cyanogenic glycosides indicated a shift from a prunasin-dominated to an amygdalin-dominated state during bitter apricot kernel development and ripening. Previous studies demonstrated that prunasin synthesized in the tegument is transported to the cotyledons via the transfer cells and converted into amygdalin in developing bitter almond seeds [42,65]. The accumulation pattern of amygdalin and prunasin in developing apricot kernels was in accord with those seen in other rosaceous stone fruits [42,64,65,66,67,68,69]. The content of cyanogenic glycosides varies among plant species, varieties, tissues and developmental stages, and can be greatly influenced by genetic factors, geographical location, seasons, soil nutrient status, cultural practices, and weather conditions [30,70,71]. In our study, since most of the examined influencing factors affecting the cyanogenic glycoside content in apricot kernels were minimized, the differences in the cyanogenic glycoside composition and content were probably determined by the genotype and ripening status of the fruits and, importantly, the differences in genetic characteristics among the apricot cultivars. The two compounds are synthesized de novo in almond seeds, and the amygdalin content is significantly higher by 200- to 1000-fold in mature bitter almond seeds than in slightly bitter or sweet seeds [66]; our results were in accord with these observations. In apricot, amygdalin concentrations of 4400 to 6500 mg/100 g DW [72] or 5500 to 7000 mg/100 g DW [8,73,74,75,76,77] have been reported; our results also fall within this range. Cyanogenic glycosides occur naturally in many plants including apricot kernels, which contain up to 6% amygdalin and are the food most likely to cause acute cyanide toxicity. This poses an ongoing risk that needs to be managed to avoid future poisoning incidents. It is reported that one-quarter of a teaspoon of ground apricot kernels or 5 to 25 apricot seeds (depending on the age and weight of the child) can suffice to cause severe cyanide intoxication [25,78]. The Agency is therefore reminding consumers that the quantities of kernels consumed per day, as set by the European Food Safety Authority (EFSA), should not exceed 1 to 3 kernels for adults and half a small kernel for young children. According to the correlation analysis between physical attributes and cyanogenic glycoside accumulation in the fruits and kernels of 10 apricot cultivars during different developmental stages, the content of prunasin in the kernel was negatively correlated with all tested indexes except for kernel moisture content (*r* = 0.47). However, the correlations between the amygdalin content of kernels and most of the tested indexes showed opposite patterns to the correlations between prunasin content and those indexes during maturity. The amygdalin content in kernels exhibited a positive correlation with kernel development stage, fruit dimension parameters (but no or a weak positive correlation with kernel dimension parameters), kernel oil content (*r* = 0.52) and β-glucosidase activity (*r* = 0.62). The systematic study of dynamic changes in cyanogenic glycosides and the activity of related enzymes during apricot kernel development periods can provide a theoretical foundation revealing the mechanism underlying apricot kernel bitter taste differentiation, and is a prerequisite for further research that may lead to the modification of cyanogen contents.

The combined effort of many investigators has led to a clear view of the biosynthesis of cyanogenic glycosides in apricots, of the tissue and subcellular organization of these compounds, and of the related genetics. In the literature, there are three theories that may explain why an almond kernel is sweet or bitter: the first is based on inherit independence (the ability to synthesize cyanogenic glycosides and β-glucosidases is inherited independently) [79,80,81]; the second is based on biosynthesis (the anabolic enzyme glucosyltransferase is responsible for the accumulation of amygdalin); and the third is based on hydrolysis by β-glucosidase (the absence of a catabolic mechanism resulting in the presence of amygdalin), which also affects kernel taste by modulating the rate of cyanogenesis [42,66,82]. However, the bitter trait is recessive [83,84], and an anabolic enzyme cannot be directly responsible for bitterness. With respect to the third possibility, a report suggested the existence of a catabolic mechanism for the control of amygdalin accumulation in almond kernels [66]. Therefore, we focused on specific catabolic enzymes, the β-glucosidases, which hydrolyze amygdalin and its precursor prunasin, resulting in the release of hydrogen cyanide. For β-glucosidase in the tested bitter kernels, a similar trend was observed between amygdalin accumulation and the changes in β-glucosidase activity from 20 to 70 or 80 DAF (Figure 3), which was consistent with the correlation analysis between them (Figure 4). The levels of amygdalin accumulation increased sharply from 50 to 70 or 80 DAF, paralleling the rapid increase in β-glucosidase activity and indicating that the increase may have occurred because the synthesis rate was much higher than the decomposition rate. Thereafter, the reduction in amygdalin levels was accompanied by a decline or a continuous increase in the levels of β-glucosidase activity, indicating that the decomposition rate may be higher than the synthesis rate at later maturity stages. Although there was no accumulation of amygdalin observed in the kernels of the sweet kernel varieties, β-glucosidase activity was still high during kernel development and ripening (Figure 4). Trace levels of stable β-glucosidase activity were even detected in the apricot sarcocarp in non-bitter cultivars, in addition to bitter-kernelled cultivars, throughout the development and ripening period (data not shown). These observations indicated that β-glucosidase was not important for the accumulation of amygdalin and its precursor prunasin in apricot kernels, and implied that (i) there might not be a single control point for amygdalin accumulation (for example, substrate supply and availability of other requisite metabolic enzymes), and (ii) to some extent, it supports the view that cyanogenic glycoside accumulation and β-glucosidase are inherited independently.

## 5. Conclusions

In this study, we systematically analyzed the dynamic changes in amygdalin and prunasin accumulation, physical characteristics (weight, moisture content, linear dimensions and derived parameters) and chemical and biochemical parameters (oil contents and β-glucosidase activity) of ten apricot cultivars during fruit and kernel development and ripening. Furthermore, correlation analysis and PCA were performed to preliminarily explore the associations between cyanogenic glycoside accumulation and pomological traits linked to quality during fruit and kernel development. High variability and significant correlations were found for most of the studied agronomic characteristics and chemical and biochemical indexes, as well as an accumulation of prunasin and amygdalin and β-glucosidase activity among the evaluated set of apricot cultivars and different ripening stages, but no or a weak positive correlation between amygdalin accumulation and kernel dimension parameters. Prunasin and amygdalin were undetectable in the sweet kernel cultivars, and were synthesized and transformed in the bitter kernels, but only amygdalin accumulated at later stages of bitter kernel maturity. The accumulation pattern of cyanogenic glycosides in bitter kernels indicated a shift from a prunasin-predominated to an amygdalin-predominated state during kernel development and ripening. The accumulated amount of cyanogenic glycosides depends on the cultivar and the state of the kernel development. β-Glucosidase was not important for amygdalin and prunasin accumulation during apricot kernel development and ripening. These findings could help us further understand the metabolic mechanisms underlying the differential accumulation of cyanogenic glycosides (amygdalin and prunasin) in bitter apricot kernels, and provide important reference values for apricot breeding (high- or low-amygdalin-content apricot cultivars), agronomic management (irrigation, fertilization, and optimum harvest time for different applications) and the intensive processing and exploitation of bitter apricot kernels (detoxification technology process).

## Figures and Tables

**Table 1:** Eigenvalues and proportion of total variability among apricot cultivars as explained by the first 10 principal components (PCs).

PC	Eigenvalue	Percentage of Variance (%)	Cumulative Contribution Rate (%)
1	11.90	54.07	54.07
2	4.69	21.32	75.39
3	2.02	9.17	84.56
4	0.93	4.24	88.80
5	0.62	2.81	91.62
6	0.58	2.62	94.24
7	0.39	1.77	96.01
8	0.32	1.45	97.46
9	0.14	0.62	98.09
10	0.11	0.51	98.59

**Table 2:** Correlations between the original variables and the first three principal components (PCs).

Variable/Factor	PC1	PC2	PC3
days after flowering (DAF)	0.27301	0.02615	−0.12214
fresh fruit weight (FWt)	0.26851	−0.1198	−0.066
fruit length (FL)	0.26229	0.03593	−0.14301
fruit width (FW)	0.27265	−0.1164	−0.01781
fruit thickness (FT)	0.26007	−0.16754	−0.05229
fruit geometric mean diameter (FGMD)	0.27327	−0.10509	−0.07017
fruit surface area (FSA)	0.27087	−0.11215	−0.06472
fruit sphericity (FSPH)	0.18928	−0.29423	0.05414
fruit shape index (FSI)	0.18351	−0.30525	0.07131
fresh kernel weight (KWt)	0.12257	0.38121	0.11151
kernel length (KL)	0.11462	0.38554	−0.18601
kernel width (KW)	0.16517	0.29583	0.1716
kernel thickness (KT)	0.18619	0.20886	0.2568
kernel geometric mean diameter (KGMD)	0.17266	0.35145	0.12211
kernel surface area (KSA)	0.18064	0.33091	0.14784
kernel sphericity (KSPH)	0.12641	−0.14565	0.56668
kernel shape index (KSI)	0.09433	−0.14983	0.60095
kernel water content	−0.25867	0.03558	0.15471
oil content	0.25584	0.01133	−0.15608
β-glucosidase activity	0.24009	0.0078	−0.14923
prunasin	−0.17993	−0.12324	−0.02838
Amygdalin	0.15883	−0.1423	−0.08524

## Data Availability

Data is contained within the article or Supplementary Materials.

## Supplementary Materials

The following are available online at https://www.mdpi.com/2304-8158/10/2/397/s1, Figure S1: Apricot fruit processing diagram. The dotted frames are two portions from fruit. The blue dotted frame represents the kernels. The red dotted frame represents the fleshy mesocarp including ectocarp, Figure S2: Length (L), width (W), and thickness (T) diagram of apricot fruits and kernels, Figure S3: Changes in geometric mean diameter (GMD) during the development of fruits and kernels in ten apricot cultivars. Values are means ± standard error (*n* = 3), Figure S4: Changes in surface area (SA) during the development of fruits and kernels in ten apricot cultivars. Values are means ± standard error (*n* = 3), Figure S5: Changes in sphericity (Sph) during the development of fruits and kernels in ten apricot cultivars. Values are means ± standard error (*n* = 3), Figure S6: Changes in shape index (SI) during the development of fruits and kernels in ten apricot cultivars. Values are means ± standard error (*n* = 3), Table S1: Changes in agronomic characteristics during the maturation of fruits and kernels in ten apricot cultivars.
