# Supplementary material for: Accumulation Pattern of Amygdalin and Prunasin and Its Correlation with Fruit and Kernel Agronomic Characteristics during Apricot (Prunus armeniaca L.) Kernel Development
Source: Foods. 2021 Feb 11;10(2):397. doi: 10.3390/foods10020397 (PMC7918717; doi:10.3390/foods10020397)
Supplement: Supplementary file 1 [file foods-10-00397-s001.pdf]

Supplementary Materials:

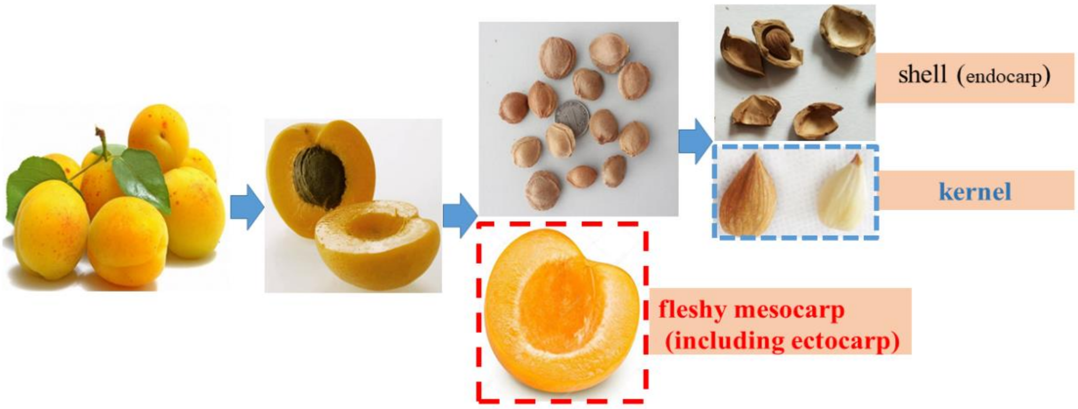

Figure S1. Apricot fruit processing diagram. The dotted frames are two portions from fruit. The blue dotted frame represents the kernels. The red dotted frame represents the fleshy mesocarp including ectocarp.

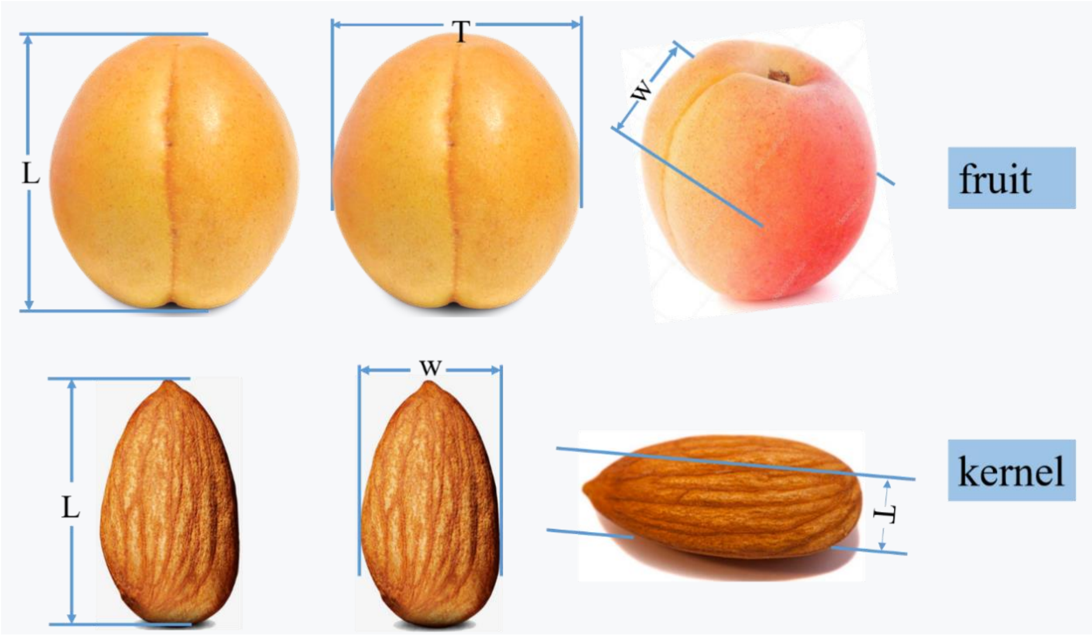

Figure S2. Length (L), width (W), and thickness (T) diagram of apricot fruits and kernels

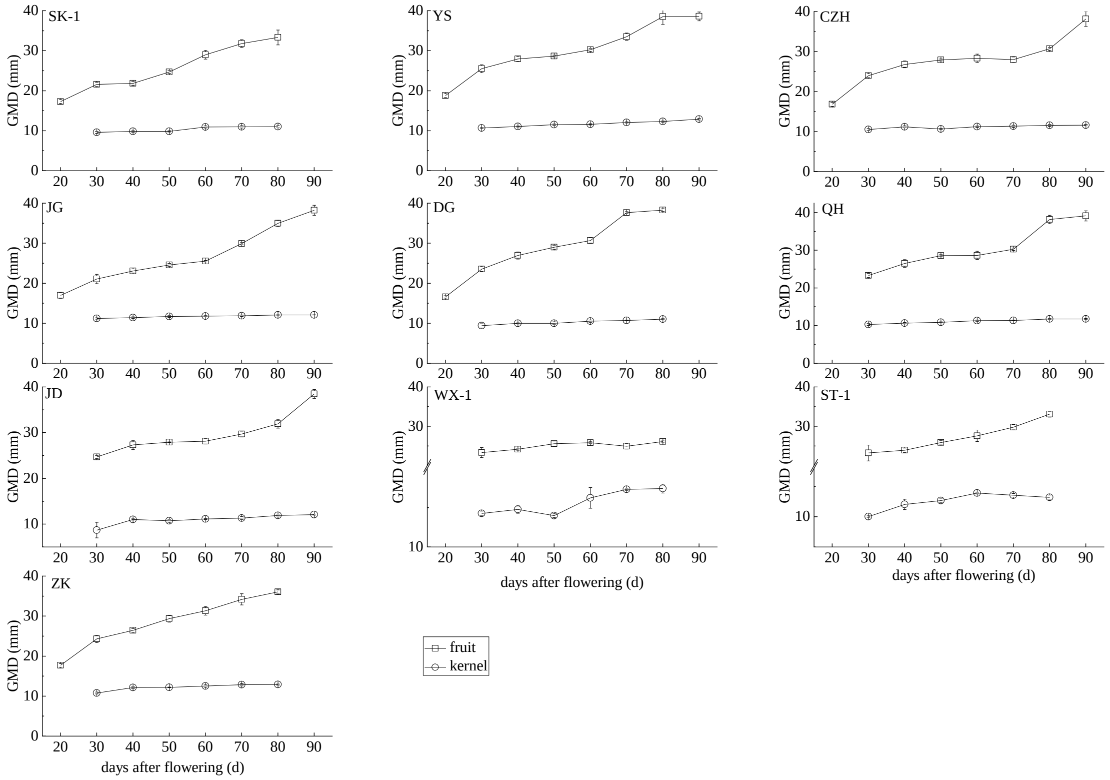

Figure S3. Changes in geometric mean diameter (GMD) during the development of fruits and kernels in ten apricot cultivars. Values are means ± standard error (n = 3).

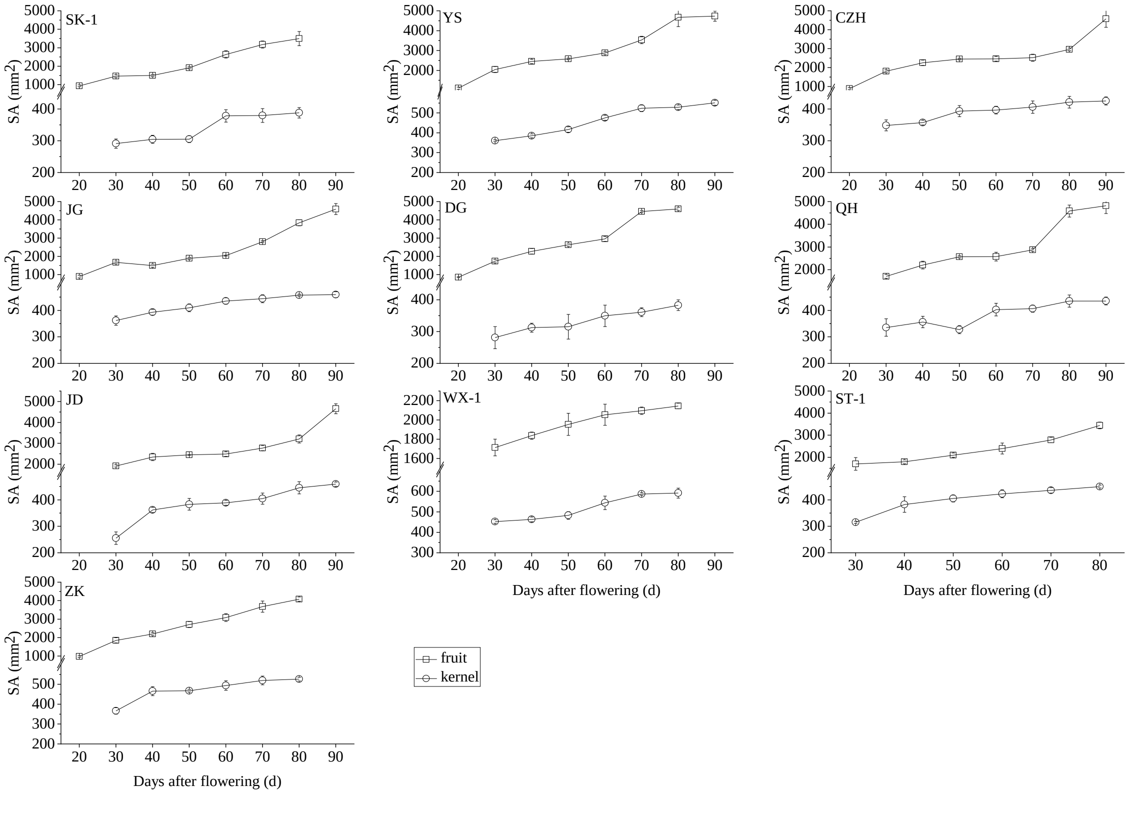

Figure S4. Changes in surface area (SA) during the development of fruits and kernels in ten apricot cultivars. Values are means ± standard error (n=3).

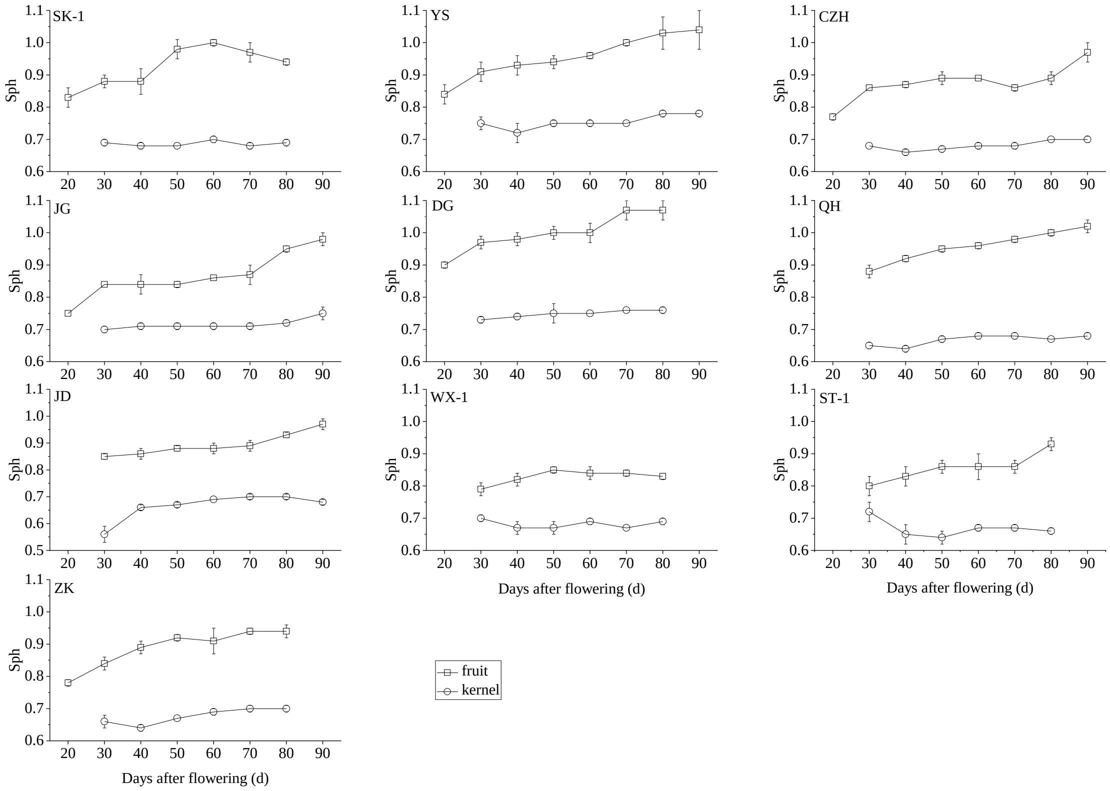

Figure S5. Changes in sphericity (Sph) during the development of fruits and kernels in ten apricot cultivars. Values are means ± standard error (n=3).

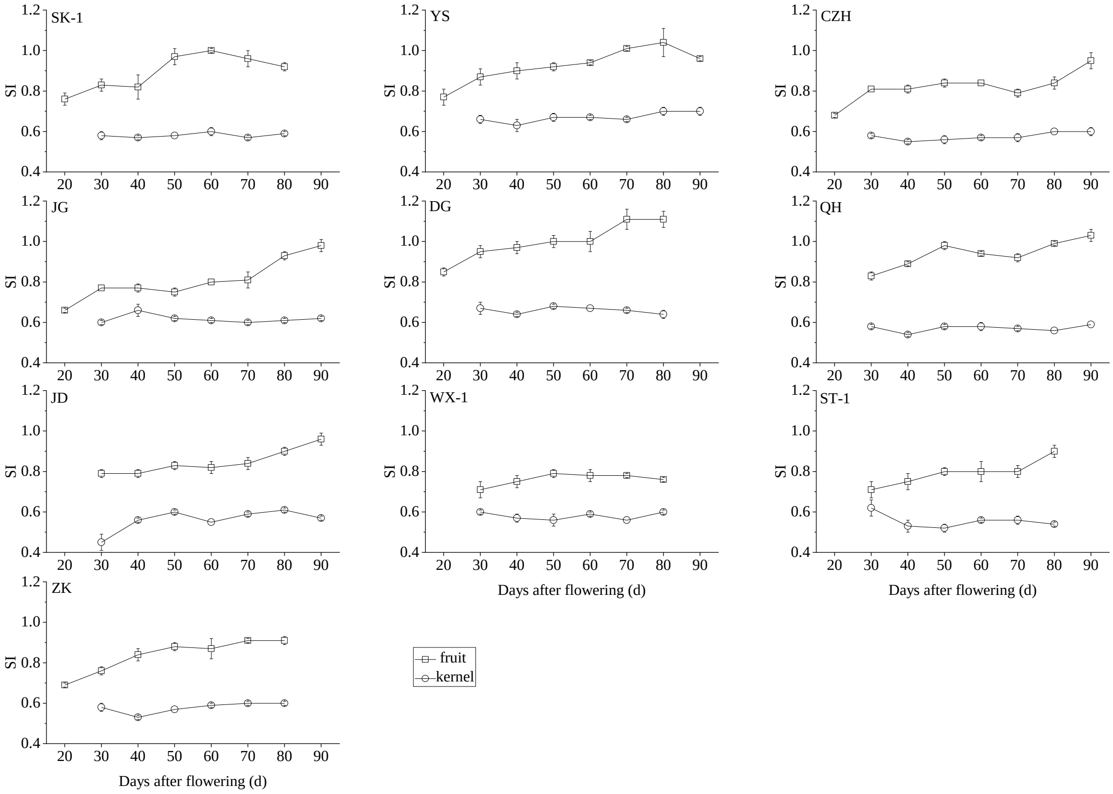

Figure S6. Changes in shape index (SI) during the development of fruits and kernels in ten apricot cultivars. Values are means ± standard error (n=3).

Table S1. Changes in agronomic characteristics during the maturation of fruits and kernels in ten apricot cultivars.

cultivar	Days after flowering（d）	L(mm)		W(mm)		T(mm)		GMD(mm)		SA(mm²)		Sph		SI	
		fruit	kernel	fruit	kernel	fruit	kernel	fruit	kernel	fruit	kernel	fruit	kernel	fruit	kernel
	20	20.81±0.06e	— —	16.75±0.38e	— —	14.91±0.4e	— —	17.32±0.48e	— —	942.79±52.09e	— —	0.83±0.03d	— —	0.76±0.03d	— —
	30	24.5±0.63d	13.99±0.49c	21.49±0.28d	9.36±0.21b	19.18±0.15d	6.81±0.22ab	21.61±0.53c	9.62±0.24b	1468.33±71.65d	291.32±14.61b	0.88±0.02c	0.69±0.01a	0.83±0.03c	0.58±0.02a
	40	24.97±0.64d	14.43±0.44c	21.53±0.93d	9.48±0.24b	19.51±0.06d	6.98±0.05ab	21.87±0.45c	9.85±0.20b	1503.28±61.51d	304.84±12.65b	0.88±0.04cd	0.68±0.01a	0.82±0.06cd	0.57±0.01a
SK-1	50	25.31±0.65d	14.48±0.29c	25.43±0.26c	10.41±0.30ab	23.45±0.30c	6.36±0.19b	24.71±0.37c	9.85±0.17b	1918.07±57.64c	305.29±10.41b	0.98±0.03ab	0.68±0.00a	0.97±0.04ab	0.58±0.00a
	60	29.09±0.59c	15.78±0.43ab	30.53±0.71b	11.67±0.68a	27.46±0.62b	7.18±0.04a	29±1.09b	10.97±0.28a	2644.59±198.46b	378.47±19.27a	1±0.01a	0.7±0.01a	1±0.01a	0.6±0.02a
	70	32.81±0.47b	16.21±0.42a	32±0.63b	11.71±0.45a	30.67±0.86a	7.33±0.29a	31.81±0.97a	10.99±0.31a	3180.31±194.89a	379.76±21.94a	0.97±0.03ab	0.68±0.01a	0.96±0.04ab	0.57±0.01a
	80	35.28±0.93a	16.19±0.55a	34.97±1.41a	11.72±0.36a	30.04±0.94a	7.35±0.26a	33.34±1.85a	11.02±0.26a	3498.96±386.63a	338.07±16.52ab	0.94±0.01b	0.69±0.01a	0.92±0.02b	0.59±0.01a
	20	22.35±0.21e	— —	18±0.4f	— —	16.5±0.44f	— —	18.79±0.49f	— —	1109.95±57.38f	— —	0.84±0.03e	— —	0.77±0.04e	— —
	30	27.99±0.44d	14.31±0.37d	25.97±0.69e	11.53±0.23d	22.87±0.74e	7.46±0.06d	25.52±0.97e	10.71±0.07f	2047.54±156.59e	360.50±4.74f	0.91±0.03d	0.75±0.02ab	0.87±0.04d	0.66±0.02ab
	40	29.92±0.28cd	15.41±0.64abc	28.5±0.84d	11.64±0.20cd	25.62±0.30d	7.58±0.22d	27.95±0.61d	11.07±0.14ef	2454.86±107.21d	385.26±9.83ef	0.93±0.03cd	0.72±0.03b	0.9±0.04cd	0.63±0.03b
YS	50	30.47±0.21c	15.32±0.48abc	29.6±0.32cd	12.22±0.08bcd	26.17±0.61d	8.17±0.19c	28.68±0.23cd	11.52±0.19de	2584.59±40.56cd	416.93±13.78de	0.94±0.02cd	0.75±0.01ab	0.92±0.02cd	0.67±0.02ab
	60	31.55±0.09bc	15.38±0.17ab	31.11±0.54c	12.45±0.18bc	28.26±0.35c	8.76±0.19b	30.27±0.37c	11.63±0.16cd	2878.8±71.08c	475.87±12.48c	0.96±0.01bcd	0.75±0.01ab	0.94±0.01bcd	0.67±0.01ab
	70	33.4±0.49b	16.29±0.17a	34.17±0.77b	12.83±0.18ab	33.07±0.35b	8.83±0.25b	33.54±0.90b	12.09±0.15bc	3536.23±187.9b	523.71±16.59b	1±0.01a	0.75±0.00ab	1.01±0.01a	0.66±0.01ab
	80	37.53±1.71a	16.86±0.32a	40.01±1.11a	12.87±0.25ab	38.16±0.99a	8.85±0.21ab	38.54±1.91a	12.31±0.16b	4673.23±463.82a	529.15±11.02b	1.03±0.05a	0.78±0.01a	1.04±0.07a	0.70±0.02a
	90	37.54±1.18a	16.90±0.20a	40.31±0.35a	12.99±0.34a	38.24±0.95a	8.95±0.15a	38.61±1.1a	12.91±0.21a	4739.31±256.35a	551.16±11.97a	1.04±0.08a	0.78±0.01a	0.96±0.01bc	0.70±0.02a
	20	22.74±0.38e	— —	16.57±0.35f	— —	14.76±0.22f	— —	17.71±0.43g	— —	986.19±47.58g	— —	0.78±0.01d	— —	0.69±0.01d	— —
	30	29.13±0.87d	15.74±0.19b	23.68±0.58e	11.27±0.51c	20.84±0.52e	7.11±0.19b	24.31±0.91f	10.8±0.23c	1858.12±139.01f	366.61±15.92c	0.84±0.02c	0.69±0.02a	0.76±0.02c	0.58±0.02a
	40	29.81±0.13cd	18.97±0.70a	25.43±0.33d	12.69±0.30b	24.41±0.81d	7.5±0.18b	26.44±0.49e	12.17±0.3ab	2197.39±81.5e	466.11±22.44ab	0.89±0.02b	0.64±0.01b	0.84±0.03b	0.53±0.01b
ZK	50	31.89±0.76c	18.26±0.20a	28.79±0.44c	13.49±0.20ab	27.63±0.37c	7.38±0.12b	29.38±0.88d	12.2±0.09ab	2713.52±161.49d	467.83±6.75ab	0.92±0.01ab	0.67±0.00ab	0.88±0.02ab	0.57±0.00ab
	60	34.47±1.28b	18.11±0.56a	30.58±0.74b	13.12±0.44ab	29.23±0.37c	8.29±0.08a	31.34±1.08c	12.54±0.30a	3088.55±214.15c	494.23±23.85a	0.91±0.04ab	0.69±0.01a	0.87±0.05ab	0.59±0.01a
	70	36.51±1.02ab	18.45±0.45a	34.1±0.49a	13.84±0.11a	32.15±0.94b	8.35±0.46a	34.21±1.42b	12.86±0.28a	3680.39±302.31b	519.92±22.28a	0.94±0.01a	0.7±0.01a	0.91±0.01a	0.6±0.01a
	80	38.44±0.52a	18.51±0.08a	34.27±0.27a	13.85±0.33a	35.63±0.40a	8.37±0.51a	36.07±0.51a	12.93±0.13a	4088.14±114.43a	526.63±9.52a	0.94±0.02a	0.7±0.01a	0.91±0.02a	0.6±0.01a
	20	21.87±0.25e	— —	15.5±0.32f	— —	14.06±0.24e	— —	16.83±0.48e	— —	890.05±50.28e	— —	0.77±0.01d	— —	0.68±0.01d	— —
	30	27.75±0.38d	15.4±0.41c	23.96±0.21e	10.91±0.43a	20.77±0.30d	6.79±0.13b	23.99±0.51d	10.53±0.26c	1808.85±76.66d	348.53±17.28c	0.86±0bc	0.68±0.00ab	0.81±0.00bc	0.58±0.01ab
	40	30.85±0.35c	16.88±0.18b	26.09±0.49d	11.16±0.39a	23.94±0.67c	7.26±0.18ab	26.81±0.87c	11.19±0.25abc	2259.36±145.31c	357.38±7.39bc	0.87±0.01bc	0.66±0.01b	0.81±0.02bc	0.55±0.01b
CZH	50	31.32±0.37c	15.91±0.16abc	27.4±0.28cd	11.22±0.24a	25.4±0.33c	6.99±0.13b	27.93±0.26c	10.66±0.11bc	2450.62±44.86c	393.45±17.4abc	0.89±0.02b	0.67±0.01ab	0.84±0.02b	0.56±0.02ab
	60	31.99±0.63c	16.01±0.29bc	28.06±0.77c	11.43±0.57a	25.37±0.45c	7.53±0.16a	28.35±1.04c	11.23±0.17abc	2467.1±117.52c	396.66±12.28abc	0.89±0.00b	0.68±0.01ab	0.84±0.00b	0.57±0.01ab
	70	32.72±0.22bc	16.63±0.14ab	27.2±0.59cd	11.75±0.10a	24.71±0.34c	7.64±0.24a	28.02±0.67c	11.36±0.28ab	2526.57±184.12c	406.05±19.59ab	0.86±0.01c	0.68±0.01ab	0.79±0.02c	0.57±0.02ab
	80	34.48±0.64b	16.82±0.55ab	30.33±0.31b	11.9±0.39a	27.7±0.29b	7.79±0.11a	30.71±0.35b	11.58±0.25a	2962.9±68.13b	421.68±18.37a	0.89±0.02b	0.7±0.00a	0.84±0.03b	0.6±0.05a
	90	38.47±2.24a	17.08±0.08a	35.36±2.75a	12.13±0.39a	34.2±3.49a	7.81±0.17a	38.17±1.86a	11.63±0.18a	4584.45±452.55a	425.3±12.83a	0.97±0.03a	0.7±0.01a	0.95±0.04a	0.6±0.02a
	20	22.5±0.53f	— —	15.6±0.47f	— —	13.88±0.47e	— —	16.95±0.69d	— —	903.37±73.16e	— —	0.75±0b	— —	0.66±0.01d	— —
	30	27.6±0.4e	15.95±0.24ab	22.82±0.08e	11.20±0.56c	19.53±0.44de	7.86±0.16bc	21.05±1.17c	11.19±0.16cd	1674.34±75.19d	361.76±17.67d	0.84±0b	0.70±0.01b	0.77±0cd	0.60±0.01b
	40	29.36±0.39d	15.24±0.70b	24.42±0.11d	11.97±0.09abc	15.58±6.4cde	7.29±0.36c	23.08±0.52cd	11.42±0.21bc	1500.04±85.5d	393.37±10.96cd	0.72±0.03ab	0.71±0.01ab	0.77±0.02dc	0.66±0.03a
	50	29.41±0.29d	15.03±0.39b	24.97±0.06d	11.29±0.47bc	20.74±0.38cde	8.09±0.10ab	24.59±0.36c	11.72±0.27bc	1899.52±55.32d	409.96±14.68bc	0.84±0.01ab	0.71±0.01ab	0.75±0.02cd	0.62±0.01ab
JG	60	29.61±0.11d	16.58±0.43a	25.15±0.19d	12.17±0.16ab	22.31±0.15cd	8.16±0.23ab	25.52±0.18bc	11.77±0.16ab	2045.68±28.25d	435.65±11.81ab	0.86±0.04ab	0.71±0.01ab	0.8±0.13bc	0.61±0.02ab
	70	34.41±0.41c	16.72±0.17a	29.03±0.56c	12.26±0.06a	26.81±0.21bc	8.18±0.29ab	29.91±0.26b	11.89±0.2ab	2811.12±49.14c	444.3±14.92ab	0.87±0.03ab	0.71±0.01ab	0.81±0.04bc	0.6±0.01b
	80	36.72±0.58b	16.73±0.39a	34.89±0.56b	12.31±0.08a	33.37±0.31ab	8.34±0.09ab	34.97±0.77a	12.07±0.05a	3842.31±169.33b	457.94±3.87a	0.95±0.01a	0.72±0.01ab	0.93±0.02ab	0.61±0.03ab
	90	38.81±0.48a	17.16±0.19a	38.49±0.97a	12.32±0.05a	37.41±0.85a	8.63±0.16a	38.23±1.25a	12.09±0.02a	4595.07±299.19a	459.42±11.34a	0.98±0.02a	0.75±0.02a	0.98±0.03a	0.62±0.01ab
	20	18.49±0.08e	— —	16.71±0.11f	— —	14.8±0.36f	— —	16.6±0.3f	— —	865.49±30.68f	— —	0.9±0.01c	— —	0.85±0.02b	— —
	30	24.39±0.25d	12.55±0.81c	23.8±0.59e	10.34±0.21b	22.51±0.44e	6.5±0.83b	23.55±0.61e	9.42±0.62b	1743.12±89.83e	281.18±34.92c	0.97±0.02b	0.73±0.01a	0.95±0.03b	0.67±0.03a
DG	40	27.53±0.29c	13.56±0.36abc	27.08±0.51d	10.25±0.26b	26.27±0.8d	7.17±0.18ab	26.95±0.9d	9.97±0.13ab	2283.89±151.79d	312.48±13.89bc	0.98±0.02b	0.74±0.01a	0.97±0.03b	0.64±0.01a
	50	29±0.63c	13.05±0.24bc	28.97±0.05c	10.57±0.17ab	29.05±0.34c	7.21±0.40ab	29±0.5c	10.01±0.36ab	2642.84±90.87c	315.37±38.67bc	1±0.02b	0.75±0.03a	1±0.03b	0.68±0.01a
	60	30.7±0.86b	14.28±0.25ab	30.95±0.43b	10.61±0.29ab	30.46±0.21b	7.72±0.27ab	30.69±0.69b	10.54±0.30a	2960.75±133.09b	349.74±33.65ab	1±0.03b	0.75±0.00a	1±0.05b	0.67±0.00a

	70	35.14±0.46a	14.37±0.49ab	38.39±0.43a	10.87±0.11ab	39.66±0.53a	7.94±0.12a	37.67±0.25a	10.72±0.12a	4459.18±58.04a	361.24±13.96ab	1.07±0.03a	0.76±0.00a	1.11±0.05a	0.66±0.01a
	80	35.69±0.65a	14.58±0.15a	38.66±0.24a	11.08±0.21a	40.69±0.24a	8.33±0.19a	38.29±0.35a	11.04±0.14a	4605.79±84.34a	383.03±16.72a	1.07±0.03a	0.76±0.01a	1.11±0.04a	0.64±0.02a
	30	26.54±0.35d	15.13±0.32c	23.61±0.49d	10.41±0.44c	20.26±0.38e	6.99±0.18abc	23.33±0.56d	10.32±0.29c	1709.86±81.88d	335.39±32.92c	0.88±0.02f	0.65±0.01a	0.83±0.02f	0.58±0.01a
	40	28.75±0.54c	16.23±0.38bc	27.11±0.76c	11.39±0.40bc	23.92±0.5d	6.42±0.05c	26.52±1.03c	10.64±0.19c	2210.96±170.26c	356.21±21.60c	0.92±0.01e	0.64±0.01b	0.89±0.01e	0.54±0.01b
	50	29.12±0.34c	16.52±0.47b	29.48±0.51b	12.08±0.18ab	26±0.57b	6.58±0.28bc	28.61±0.27b	10.89±0.13bc	2571.75±48.31b	372.44±15.42bc	0.95±0.01c	0.67±0.01ab	0.98±0.02d	0.58±0.01a
**QH**	60	30.29±0.83b	16.77±0.33ab	29.9±0.58b	12.12±0.33ab	27.3±0.5c	6.92±0.12abc	28.66±1.09b	11.33±0.19ab	2582.9±193.63b	403.18±23.68ab	0.96±0.01bc	0.68±0.01a	0.94±0.01cd	0.58±0.02a
	70	31.54±0.17bc	16.62±0.26b	31.42±0.34b	12.28±0.64ab	28.03±0.13bc	7.18±0.19ab	30.28±0.31b	11.38±0.11ab	2881.3±59.25b	406.82±13.53ab	0.98±0.01b	0.68±0.01a	0.92±0.02bc	0.57±0.01a
	80	38.35±0.44a	17.29±0.62ab	38.73±1.12a	12.83±0.30a	37.55±0.47a	7.25±0.28a	38.2±1.11a	11.77±0.18a	4588.01±266.05a	435.37±23.15a	1±0.01ab	0.67±0.00ab	0.99±0.01ab	0.56±0.04ab
	90	39.16±0.53a	17.95±0.18a	39.46±0.54a	13.15±0.37a	39.31±1.01a	7.36±0.14a	39.15±1.37a	11.78±0.12a	4819.03±339.4a	435.68±14.83a	1.02±0.02a	0.68±0.01a	1.03±0.03a	0.59±0.03a
	30	29.06±0.5d	15.35±0.70b	24.27±0.23e	7.78±3.03b	21.44±0.15e	6.38±0.13d	24.73±0.33e	8.70±1.70b	1921.14±51.05e	255.63±23.61b	0.85±0.01d	0.56±0.03b	0.79±0.02d	0.45±0.09b
	40	31.96±0.31c	16.62±0.36ab	26.45±0.53d	11.27±0.13ab	24.18±0.83d	7.20±0.08abc	27.34±1d	11.05±0.13a	2349.79±173.59d	362.18±22.05ab	0.86±0.02d	0.67±0.01ab	0.79±0.02d	0.56±0.01ab
	50	31.73±0.24c	15.30±0.51b	27.67±0.3d	11.36±0.13ab	24.84±0.14d	7.11±0.34bc	27.93±0.07d	10.73±0.33ab	2451.55±12.09d	383.39±9.37a	0.88±0.01cd	0.66±0.00a	0.83±0.02cd	0.60±0.01a
**JD**	60	32.08±0.08c	15.91±0.36b	27.78±0.59d	11.69±0.64a	25.04±0.45cd	7.01±0.15c	28.15±0.61d	11.13±0.13a	2491±108.99d	389.05±8.87a	0.88±0.02cd	0.69±0.01a	0.82±0.03cd	0.55±0.00ab
	70	33.49±0.86bc	17.61±0.05a	29.62±0.4c	12.12±0.21a	26.44±0.23c	7.48±0.06abc	29.71±0.65c	11.34±0.30a	2773.97±122.19c	404.87±21.28a	0.89±0.02c	0.70±0.00ab	0.84±0.28c	0.59±0.01a
	80	34.3±0.44b	18.02±0.34a	31.55±0.77b	12.37±0.37a	30.19±0.6b	7.72±0.32ab	31.97±0.98b	11.90±0.31a	3212.62±196.64b	445.45±23.08a	0.93±0.01b	0.70±0.01a	0.9±0.02b	0.61±0.01a
	90	39.68±0.83a	16.72±0.56ab	38.37±0.38a	12.9±0.15a	37.46±0.67a	7.79±0.17a	38.49±0.97a	12.09±0.10a	4656.65±236.36a	459.39±7.93a	0.97±0.02a	0.68±0.01a	0.96±0.03a	0.57±0.01a
	30	29.41±0.31c	17.43±0.27c	23.35±0.92c	13.42±0.24a	18.51±0.85c	7.65±0.08ab	23.33±1.27c	12.14±0.15bc	1713.79±86.98c	452.85±8.87c	0.79±0.02b	0.70±0.01a	0.71±0.04b	0.60±0.01a
	40	29.39±0.36c	18.66±0.71abc	24.97±0.2bc	13.79±0.26a	19.3±0.66bc	7.42±0.37b	24.18±0.25bc	12.39±0.24bc	1837.45±38.37bc	463.31±11.46bc	0.82±0.02ab	0.67±0.02a	0.75±0.03ab	0.57±0.02a
**WX-1**	50	29.61±0.63bc	18.04±0.88bc	25.7±1.18b	12.34±0.17a	20.08±0.15abc	7.79±0.13ab	25.57±0.68a	12.00±0.12c	1954.76±114.84ab	482.78±18.63bc	0.85±0.01a	0.67±0.02a	0.79±0.02a	0.56±0.03a
	60	30.09±0.25bc	18.95±1.22abc	26.66±0.23a	14.12±0.70a	20.37±0.37ab	8.46±0.32ab	25.83±0.24a	13.13±0.66ab	2054.78±109.98a	544.27±32.87ab	0.84±0.02a	0.69±0.01a	0.78±0.03a	0.59±0.01a
	70	30.76±0.43ab	19.89±0.17ab	26.71±0.33a	14.18±0.24a	20.79±0.35ab	8.87±0.27a	25.94±0.73a	13.67±0.07a	2096.43±39.35a	587.32±6.06a	0.84±0.01a	0.67±0.00a	0.78±0.01a	0.56±0.00a
	80	31.38±0.15a	20.52±0.30a	26.77±0.24a	14.38±0.16a	21.42±0.46a	8.94±0.10a	26.13±0.18a	13.72±0.28a	2145.76±29.06a	591.78±24.06a	0.83±0.01a	0.69±0.01a	0.76±0.01a	0.60±0.01a
	30	29.09±0.93c	15.48±1.21a	21.62±1.23e	10.49±0.4ab	19.92±1.25c	6.67±0.31b	23.22±2.01e	10.02±0.06c	1701.8±286.26e	315.47±3.89c	0.8±0.03c	0.72±0.04a	0.71±0.04c	0.62±0.07a
	40	28.97±0.28c	15.75±0.57a	22.63±0.62de	10.80±0.54a	20.88±0.42c	7.76±0.45ab	23.91±0.58de	11.02±0.43b	1797.47±86.97de	382.5±29.29b	0.83±0.03bc	0.65±0.03a	0.75±0.04bc	0.53±0.03a
**ST-1**	50	30.13±0.17bc	17.47±0.14a	24.18±0.21cd	9.63±0.41b	23.75±0.78bc	8.04±0.09a	25.86±0.59cd	11.35±0.18ab	2101.61±95.35cd	405.22±13.13ab	0.86±0.02b	0.64±0.02a	0.8±0.02b	0.52±0.02a
	60	32.08±1.01b	17.60±0.86a	25.27±0.76c	11.31±0.06a	26.14±2.52b	8.23±0.73a	27.6±1.45c	11.97±0.07a	2397.48±249.25c	422.96±14.75ab	0.86±0.04b	0.67±0.01a	0.8±0.07b	0.56±0.01a
	70	34.75±1a	17.63±0.52a	28.53±0.16b	11.39±0.20a	26.65±0.27b	8.32±0.27a	29.78±0.68b	11.78±0.14a	2786.79±125.65b	436.31±10.20a	0.86±0.02b	0.67±0.01a	0.8±0.03b	0.56±0.02a
	80	35.43±0.63a	17.8±0.39a	31.71±0.63a	11.01±0.24a	32.29±0.5a	8.52±0.14a	33.1±0.74a	11.60±0.20ab	3443.08±153.97a	450.14±5.41a	0.93±0.02a	0.66±0.01a	0.9±0.03a	0.54±0.01a

Values are means ± standard error (n=3). Different letters corresponding to the different development stages for the same cultivars in the same column show significant differences according to ANOVA ($p<0.05$); Apricot cultivars: Shanku-1 (SK-1), Cuanzhihong (CZH), Zhengkui (ZK), Jiguang (JG), Jidan (JD), Yangshao (YS), Qiuhong (QH), Daguo (DG), Shantian-1 (ST-1), Weixuan-1 (WX-1); Wt: weight, L: length, W: width, T: thickness, GMD: geometric mean diameter, SA: surface area, Sph: sphericity, SI: shape index.
